# Assessment of the Coronary Venous System Using 256-Slice Computed Tomography

**DOI:** 10.1371/journal.pone.0104246

**Published:** 2014-08-04

**Authors:** Chunjuan Sun, Yinghua Pan, Hongbo Wang, Jian Li, Pei Nie, Ximing Wang, Heng Ma, Futao Huo

**Affiliations:** 1 Shandong Provincial Key Laboratory of Diagnosis and Treatment of Cardio-Cerebral Vascular Diseases, Shandong Medical Imaging Research Institute, Shandong University, Jinan, Shandong, China; 2 Department of Radiology, Yuhuangding Hospital, Yantai, Shandong, China; University of Iowa Carver College of Medicine, United States of America

## Abstract

**Purpose:**

To investigate the coronary venous system and its relation to adjacent structures using 256-slice computed tomography (CT).

**Materials and Methods:**

The study consisted of 102 patients who underwent coronary CT angiography (CTA) using 256-slice CT. For each patient, the coronary venous system and its relation to adjacent structures were evaluated. The appropriate locations and diameters of the posterior interventricular vein (PIV), posterior vein of the left ventricle (PVLV) and the left marginal vein (LMV) were detected. The paired student's *t* test was used to evaluate the difference between the diameter of the coronary sinus (CS) ostium in anteroposterior direction and that in superoinferior direction.

**Results:**

The CS, great cardiac vein (GCV), PIV, and anterior interventricular vein (AIV) were visualized in all cases. It was possible to evaluate at least one main vein with adequate caliber and regular course for cardiac resynchronization therapy (CRT) in 96.1% of these cases. The diameter of the CS ostium in superoinferior direction (11.7±2.1 mm) was larger than that in anteroposterior direction (9.0±2.0 mm) (t = 13.511, P<0.05). For the majority of the cases, the CS-GCV was located above the level of the mitral valve annulus (MVA), while the left circumflex coronary artery (LCX) was coursed between the CS-GCV and the MVA. LMV had more intersection with the circumflex or circumflex marginal than PVLV.

**Conclusion:**

256-slice CT provides superior noninvasive evaluation of the coronary venous system which has important clinical implications.

## Introduction

Investigations of the coronary venous system are overshadowed by numerous studies of the coronary arteries. In contrast to that for coronary arteries, the literature for coronary veins is scarce. The clinical importance of the coronary venous system, nevertheless, should not be underestimated [1].

The coronary venous system is being used increasingly for various electrophysiological purposes, such as radiofrequency catheter ablation (RFA), mapping, defibrillation, local drug delivery, gene delivery, percutaneous mitral annuloplasty (PMA) and cardiac resynchronization therapy (CRT) [2], [3]. A thorough knowledge of the coronary venous anatomy prior to the procedure will not only ease the intervention process but also increase the success rate. Catheter-based venous angiography is an invasive method with the possibilities for serious complications. In recent years, ECG-gated multi-slice spiral computed tomography (MSCT) has become an important noninvasive tool to evaluate coronary venous system. Up to now, a number of previous studies have used 4-, 16-, 64-, and128-slice MSCT to describe the coronary venous tree [4]–[10]. Compared with the older generation MSCT, the 256-slice MSCT with the reduction of scanning time, breath-holding duration, motion artifacts, contrast medium and radiation dose can provide superior noninvasive visualization of coronary artery and venous anatomy [11]. However, to our best knowledge, there are no published papers regarding coronary venous system depiction using the latest generation 256-slice CT.

The aim of our study was to assess coronary venous system and its relation to adjacent structures using 256-slice CT.

## Materials and Methods

Our study received the approval from Shandong Medical Imaging Research Institute Ethics Board. Our institutional review board waived the need for written informed consent from the participants, because it was a retrospective review of coronary CT angiography performed for clinical reasons.

### Patients

The study population was composed of 102 consecutive patients (55 females and 47 males; mean age: 58.8±8.8 years, range: 28–79years) who had undergone coronary CTA between July 2013 and November 2013 for suspected coronary artery disease. Patients with a creatinine level above 2 mg/dL, pregnancy and any known allergy to non-ionic contrast agents were excluded from the study.

### CT protocol

Imaging was performed with a retrospectively ECG-gated CT coronary angiogram using 256-slice CT (Brilliance iCT, Philips, Cleveland,Ohio,USA). The scan coverage was planned from the tracheal bifurcation to the diaphragm. The coronary CTA scan parameters were as follows: tube voltage, 100 kVp; effective tube current–time product, 700 mAs; pitch, 0.18; detector configuration, 128×0.625 mm; rotation time, 270 ms. The heart rate was controlled under 90 beats per minute (bpm) by β-blocker prior to CTA. On average, 75 ml of non-ionic contrast agent (Ultravist 370, Bayer Schering Pharma AG, Berlin, Germany) was injected into the antecubital vein at a rate of 5 ml/s using a dual-head injector. The contrast agent was given in 3 phases: 60 ml of contrast agent (average), then 15 ml of contrast agent and 15 ml of saline (50%/50%) and finally 30 ml of saline flush. Automatic bolus tracking (Bolus Pro, Philips Healthcare, Cleveland, OH, USA) was used by defining a region of interest (ROI) in the ascending aorta at the level of aorto-pulmonary fenestration, with the scans initiated 6 s after the signal attenuation reached a pre-determined threshold of 180 Hounsfield Units (HU).

Data reconstruction was performed on a dedicated workstation (IntelliSpace Portal, Cleveland,Ohio,USA). Transverse images were reconstructed at 45% of the RR interval with 0.9 mm slice thickness and 0.6 mm intervals using a medium smooth-tissue convolution kernel (B26f). Multiplanar reformation (MPR), maximum intensity projection (MIP) and volume rendering (VR) were used for image interpretation. All images were interpreted by two independent radiologists with more than 6-year cardiac CT experience. Consensus agreement was achieved between the two observers if disagreement existed.

### Anatomic observations

**Figure 1:**
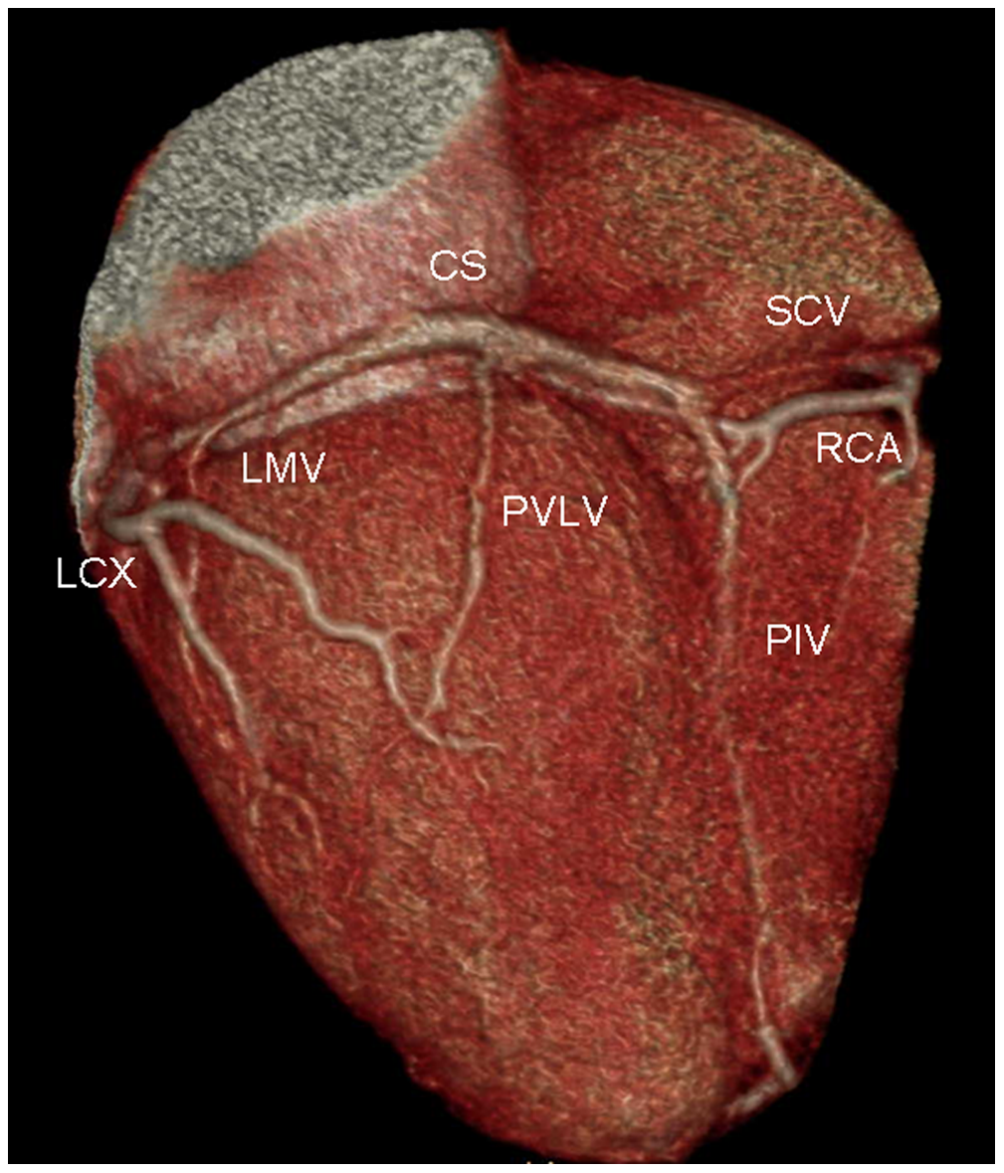
Volume-rendered image provides an overview of coronary venous anatomy. It clearly depicts the coronary sinus (CS), posterior interventricular vein (PIV), posterior vein of the left ventricle (PVLV), left marginal vein (LMV), and small cardiac vein (SCV). Also notes the right coronary artery (RCA) and the left circumflex coronary artery (LCX).

The tributaries of the coronary venous system and its relation to adjacent structures were identified on volume-rendered images (Figure 1). The presence of the following coronary veins was evaluated: CS, GCV, AIV, PIV, PVLV, LMV, small cardiac vein (SCV) and the vein of Marshall. The number of side branches of these tributaries was also recorded.

### Quantitative data

**Figure 2:**
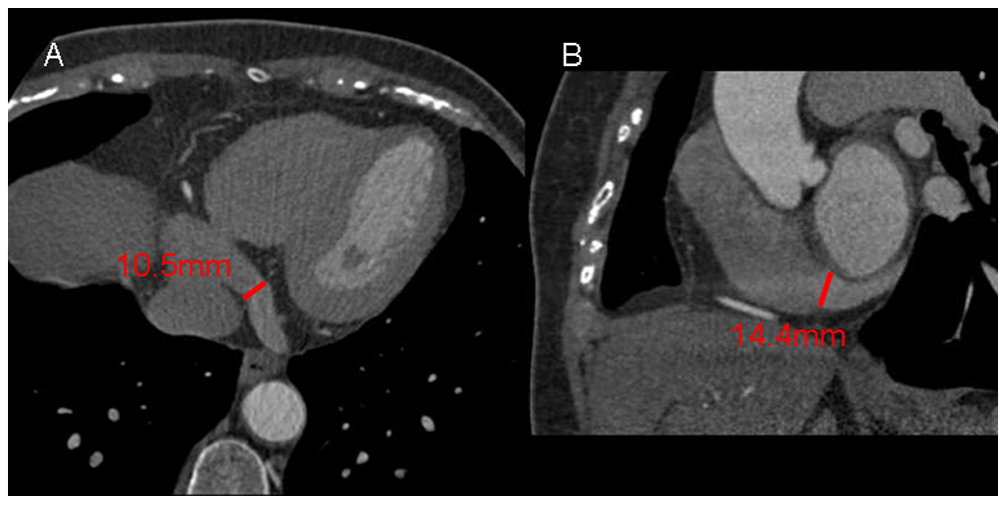
Measurement of the diameter of the coronary sinus (CS). Multiplanar reformatting is used to determine the diameter of the ostium in the anteroposterior(A) and supero-inferior (B)position.

**Figure 3:**
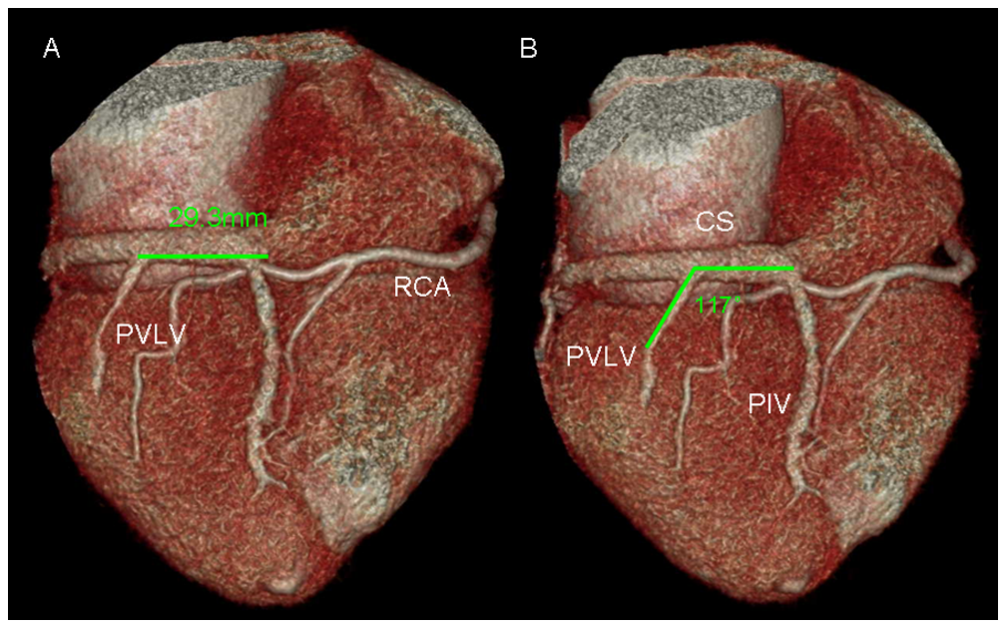
Measurement of the distance and angle between the tributaries and coronary sinus (CS). A. the distance from the ostium to tributaries B, the angle between the tributaries and CS.

The ostium of the CS is defined as the site where the CS makes an angle with the right atrium in the crux cordis area. Multi-planar reformatting was used to determine the size of the ostium in 2 directions (Figure 2). The diameters of the proximal parts of the PIV, PVLV, and LMV were measured. The distance from the ostium to tributaries and the angle between the tributaries and CS were measured on volume-rendered images (Figure 3).

### Statistical analysis

The statistical SPSS 17.0 software (SPSS, Chicago, IL, USA) was used for statistical analysis. Continuous variables were expressed as mean ± standard deviation. Categorical variables were expressed as absolute number (percentage). The paired student's *t* test was used to evaluate the difference between the diameter of the CS ostium in anteroposterior direction and that in superoinferior direction. Statistical tests were 2-tailed, and a P value<0.05 was considered significant.

## Results

### The Anatomy of coronary venous system

The coronary sinus (CS) is the most prominent feature of the coronary venous system running along the posterior coronary groove to the right atrium. It was visualized in all cases (100%).

The great cardiac vein (GCV) terminates in the coronary sinus, a junction defined by the presence of the Marshall vein. It was visualized in all cases (100%).

The anterior interventricular vein (AIV) originates at the apex of the heart, returns back along the anterior inter-ventricular groove, and ends in the great cardiac vein. It was visualized in all cases (100%).

The posterior interventricular vein (PIV) originates close to the apex of the heart and follows the posterior inter-ventricular groove toward the base where, in most cases, flows into the CS. It was visualized in all cases (100%).

The posterior vein of the left ventricle (PVLV) runs through the diaphrag-matic aspect of the left ventricle and ends in the GCV. It was visualized in 79 cases (77.5%). There were 2 PVLVs in 10 cases (12.7%).

The left marginal vein (LMV) runs along the lateral border of the heart ending in the GCV. It was visualized in 68 cases (66.7%). There were 2 or 3 LMVs in 8cases (11.8%).

The small cardiac vein (SCV) originates on the front side of the heart and runs on the diaphragmatic portion of the right coronary groove. It was observed as a very small vein in 28 cases (27.5%), and 57.1% of which directly drained into PIV.

The Marshall vein descends obliquely along the back surface of the left atrium up to the confluence of the CS and GCV. It was present in 7 cases (6.9%).

Table 1 lists the quantitative data of the PIV, PVLV, and LMV. It was possible to evaluate at least one main vein with adequate caliber and regular course for CRT in 96.1% (98/102) of the cases. The diameter of the CS ostium in superoinferior direction (11.7±2.1 mm) was larger than that in anteroposterior direction (9.0±2.0 mm). (t = 13.511, P<0.05).

**Table 1:** Quantitative measurement of cardiac veins.

	Ostial diameter (mm)	Distance from the ostium of CS (mm)	Angle between the identified veins and CS/GCV
PIV (n = 102)	4.8±1.0	10.6±4.0	71°±17°
PVLV (n = 79)	3.1±1.0	27.8±9.3	109°±18°
LMV (n = 68)	2.7±0.7	57.3±15.1	116°±29°

CS, coronary sinus; PIV, posterior interventricular vein; PVLV, posterior vein of the left ventricle; LMV, left marginal vein; GCV, great cardiac vein.

### Relationship between CS-GCV, MVA and LCX (Figures 4–5)

**Figure 4:**
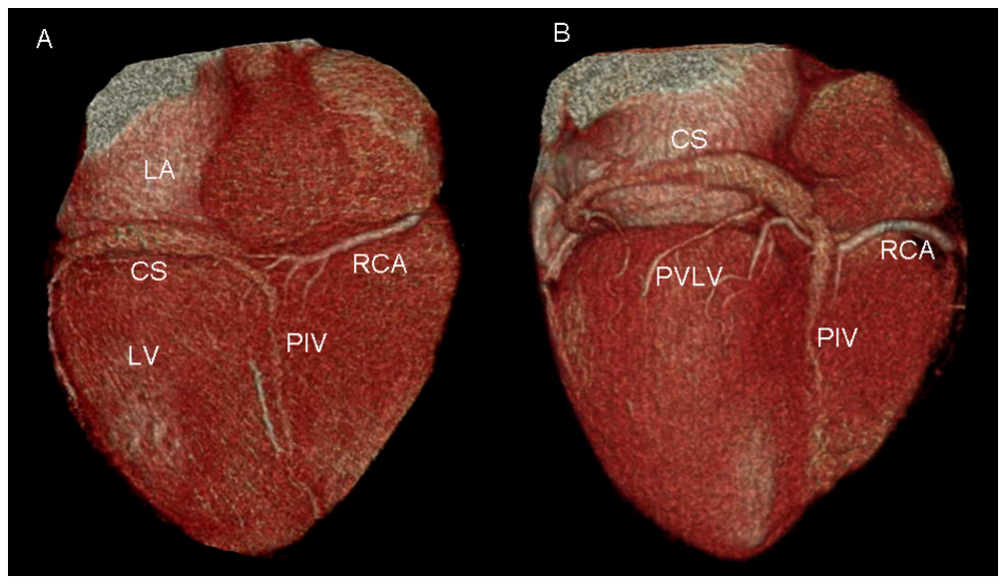
Volume-rendered images show the relationship between the coronary sinus (CS) and mitral valve annulus (MVA). A. the CS is located along the MVA. B. the CS is located above the level of the MVA.; LA = left atrium; LV = left ventricle.

**Figure 5:**
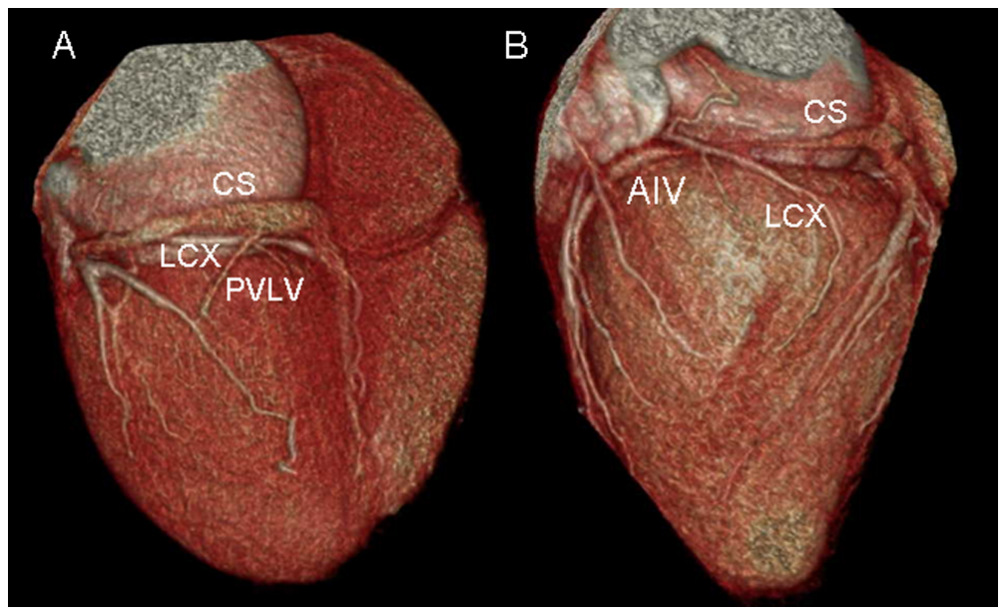
Volume-rendered images show the relationship between the coronary sinus (CS) and the left circumflex coronary artery (LCX). A. LCX courses between the CS and the mitral valve annulus (MVA), with a potential risk of compression of the LCX when percutaneous mitral annuloplasty (PMA) is applied. B. LCX courses superiorly to the CS.

The CS-GCV was located above the level of the MVA in 93.1% (95/102) of cases, and only 6.9% (7/102) of cases was located along the MVA. The relationship between LCX and CS-GCV was defined by the medial/lateral orientation with respect to determination of the vessel closer to epicardium in overlapping segments. The CS-GCV was lateral to the LCX in 68.6% (70/102) of cases and medial to the LCX in 29.4% (30/102) of cases. The CS-GCV was medial in one segment and lateral in another segment in 2 cases.

### Relationship between AIV and the left anterior descending artery (LAD) (Figure 6)

**Figure 6:**
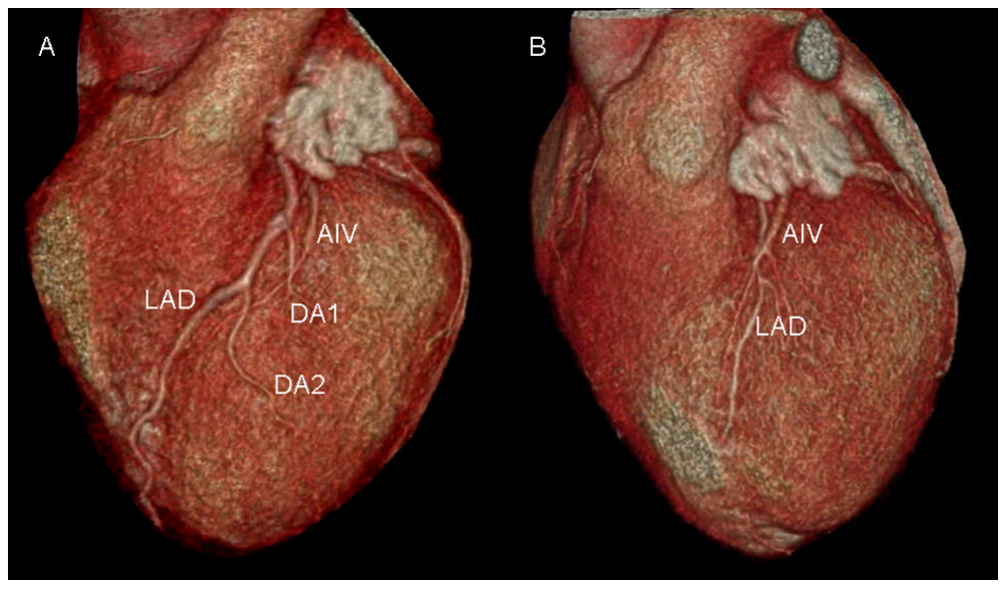
Volume-rendered images show the relationship between anterior interventricular vein (AIV) and left anterior descending artery (LAD). A. AIV is concomitant with LAD, and AIV crosses two diagonals with the vein medial to the diagonals (between epicardium and arteries). B. AIV crosses LAD, and the vein is lateral to and therefore overlying the LAD.

In 29.4% (30/102) of cases, AIV crossed LAD. In these 30 cases, the vein was lateral to and therefore overlying the LAD in 53.3% (16/30), and medial to the LAD in 46.7% (14/30). In the remaining cases (70.6%), AIV was concomitant with LAD. AIV crossed a diagonal with the vein lateral and therefore overlying the artery in 33.3% (34/102) of cases, and the vein medial to the diagonal (between epicardium and artery) in 66.7% (68/102) of cases.

### Relationship between PVLV, LMV and circumflex or circumflex marginal (Figure 7)

**Figure 7:**
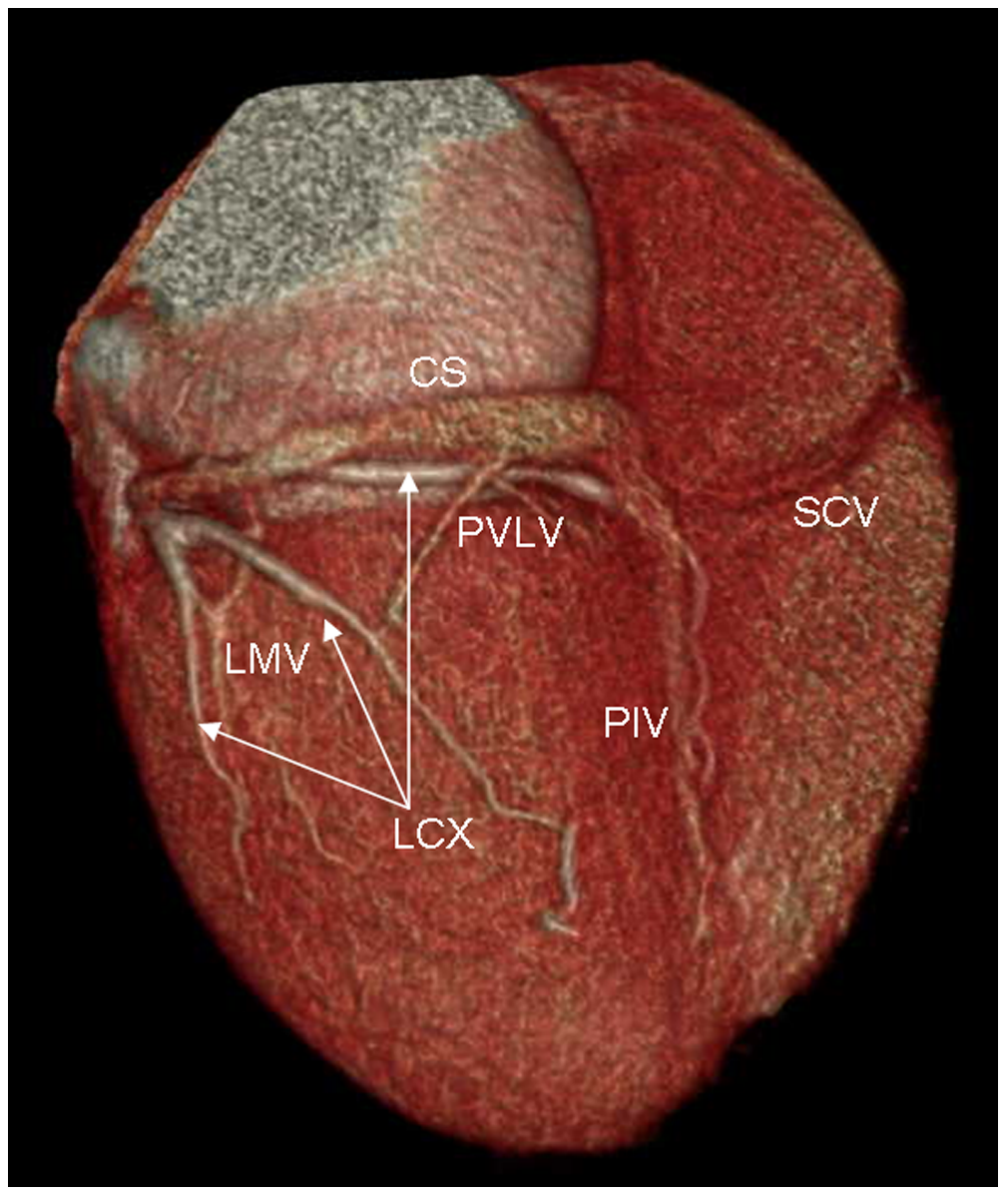
Volume-rendered image shows the relationship between posterior vein of the left ventricle (PVLV), left marginal vein (LMV) and circumflex or circumflex marginal. The LMV and PVLV cross the circumflex or circumflex marginal.

The LMV crossed the circumflex or circumflex marginal in 82.4% (56/68) of cases. The LMV was lateral to the circumflex or circumflex marginal in 67.9% (38/56) of cases, and medial to the circumflex or circumflex marginal in 32.1% (18/56) of cases. The LMV was concomitant with circumflex or circumflex marginal in 7.4% (5/68) of cases. Because of the decrease in size of the circumflex, there was an intersection point between the PVLV and circumflex marginal branches only in 32.9% (26/79) of cases.

### Relationship between PIV and posterior descending artery (PDA) (Figure 8)

**Figure 8:**
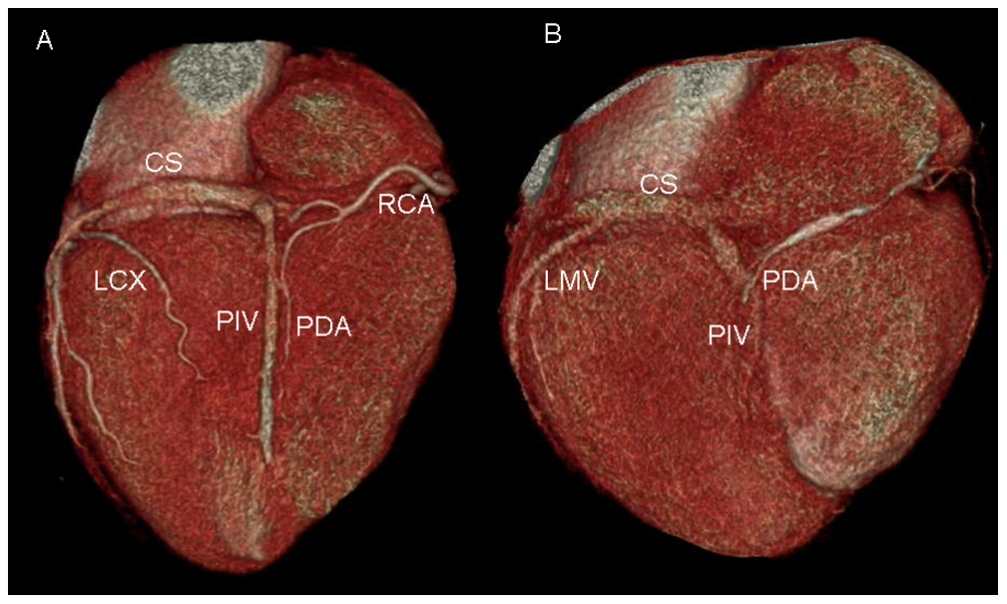
Volume-rendered images show the relationship between posterior interventricular vein (PIV) and posterior descending artery (PDA). A. PIV is concomitant with PDA. B. PIV crosses PDA with the vein medial to the PDA.

PIV was concomitant with PDA in 35 cases and intersected with PDA in 63 cases. Of these intersected cases, the vein was lateral to the PDA in 92.1% (58/63) of cases, and medial to the PDA in 7.9% (5/63) of cases.

## Discussion

### Imaging methods for coronary venous system

There are several imaging methods to assess coronary venous system. Each of these methods has its own advantages and disadvantages [12]–[17].

Retrograde cardiac venography is currently the gold standard technique for defining coronary venous anatomy; however, it is invasive, technically challenging, and unable to define the relationship between the coronary veins and coronary arteries. It also has a lot of disadvantages such as coronary sinus trauma due to balloon occlusion, a longer imaging time, and a high dose of contrast material. Compared to retrograde cardiac venography, electron-beam CT is a better option, as it is noninvasive and has a higher imaging success rate. However, a considerable number of patients may not be adequately assessed by this modality because not all parts of the heart are examined, and the image quality is suboptimal. Compared with other techniques for imaging the cardiac venous system, MRA is noninvasive and does not require injection of iodinated contrast medium or expose patients to ionizing radiation. However, the major challenges for MRA are the long scan time, image artifacts caused by motion instability during the long scan time and a relatively low spatial resolution.

In recent years, MSCT has become the preferred noninvasive method to assess the coronary venous system. Unlike retrograde venography, MSCT angiography allows both simultaneous imaging of the coronary veins and assessment of arteriovenous relationships. There is no vessel overlap. The newer-generation MSCT scanner, especially the 256-slice CT with faster gantry rotation time and larger detector coverage can provide better visualization of the coronary veins with a lower radiation dose.

### Main findings

The followings are our main findings in this study. First, to the best of our knowledge, this is the first report to assess the coronary venous system using 256-slice CT. Second, the 256-slice CT coronary angiography, in addition to coronary artery imaging, can provide important information of the coronary venous system, as well as its relation to adjacent structures. The latter has important clinical implications. Third, this kind of study can provide guidance for patients who need device implantation.

### Clinical implications

**Figure 9:**
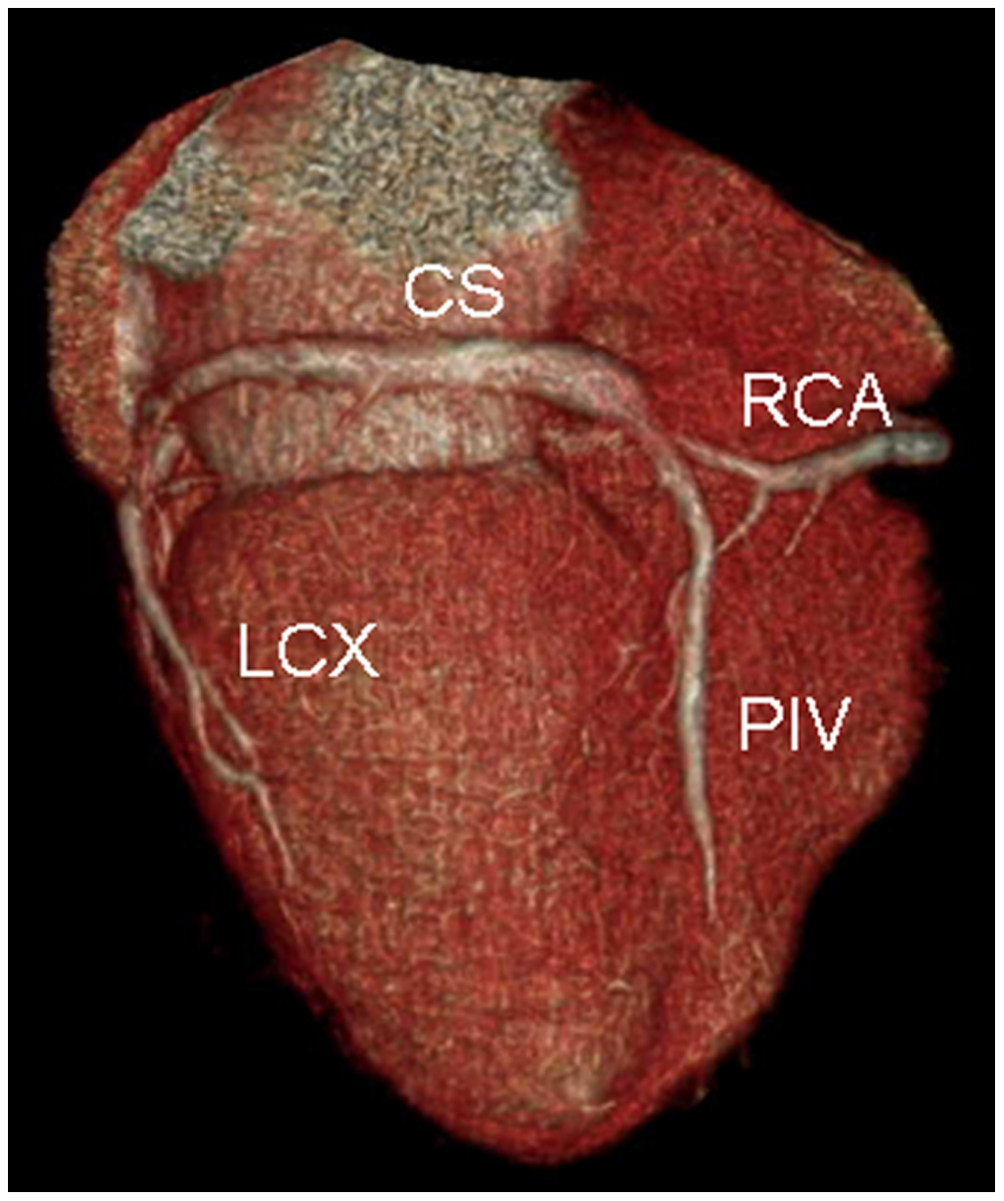
A case with no target veins for Cardiac resynchronization therapy (CRT). Volume-rendered image shows the presence of multiple thin and short posterior cardiac veins representing a contraindicated site for CRT leads placement.

Cardiac resynchronization therapy (CRT) is an attractive treatment for selected heart failure patients. Left ventricular (LV) pacing is established by a pacemaker lead in a tributary of the CS during CRT. The LV lead is the most challenging part of CRT implantation. Because the postero-lateral left ventricular wall is usually the optimal location for pacing [18]. Common target veins are the LMV or PVLV [19]. Vessel size is an important criterion for selecting the target vein for LV pacemaker lead insertion. A too-small coronary sinus diameter and a too-small or too-big target vein may cause CRT to be unsuccessful [20]. The diameter of target vein for the smallest LV lead must be greater than 1.5 mm [19]. Before CRT implantation, the following question has to be answered: which cardiac vein is suitable for LV lead placement? 256-slice CT is a reliable technique to depict the coronary venous system, which can not only confirm the presence of a target vein but also provide information on the course of the vessel, the side branches, the diameter, the distance from the ostium to tributaries, the angle between the tributaries and CS and the relation to adjacent artery. In our study, the PVLV was visualized in 77.5% of all cases, and the LMV was visualized in 66.7% of all cases. The mean diameter of PVLV and LMV was 3.1±1.0 mm, and 2.7±0.7 mm, respectively. It was possible to evaluate at least one main vein with adequate caliber and regular course running on the postero-lateral wall of left ventricle in 96.1% of the cases, only 4 cases had no target veins (Figure 9). The CS is the most constant component of the coronary venous system and was detected in all patients. The diameter of the CS ostium in superoinferior direction(11.7±2.1 mm) was larger than that in anteroposterior direction (9.0±2.0 mm), indicating an oval shape of the ostium, which is confirmed by the 16-slice MSCT observations of Jongbloed et al [5] and magnetic resonance imaging observations of Ma H et al [17]. In addition, LMV had more intersection with the circumflex or circumflex marginal than PVLV and the LMV was lateral to the circumflex or circumflex marginal in most cases. Circumflex artery compression may be caused as a serious complication with such a close relation between LMV and circumflex artery during CRT. A thorough knowledge of the coronary venous system anatomy prior to the procedure will ease the intervention process and increase the success rate.

**Figure 10:**
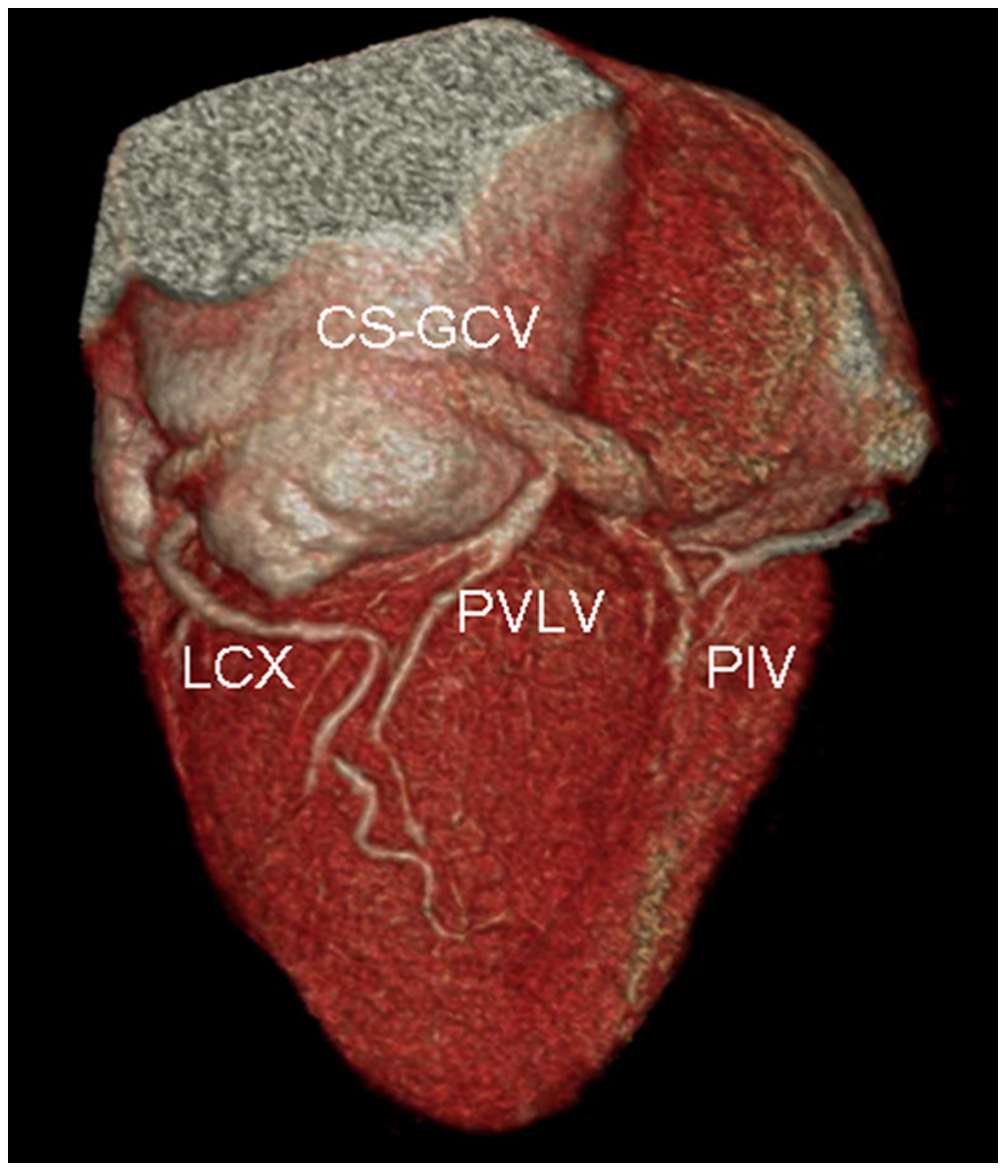
Volume-rendered image shows the spatial relationship of the coronary sinus (CS) and left atrial wall. In this patient, the CS lies on the left atrial wall for almost its entire course and part of the CS is not visualized because of the compression of left atrial wall, which may limit the use of Percutaneous mitral annuloplasty (PMA).

**Figure 11:**
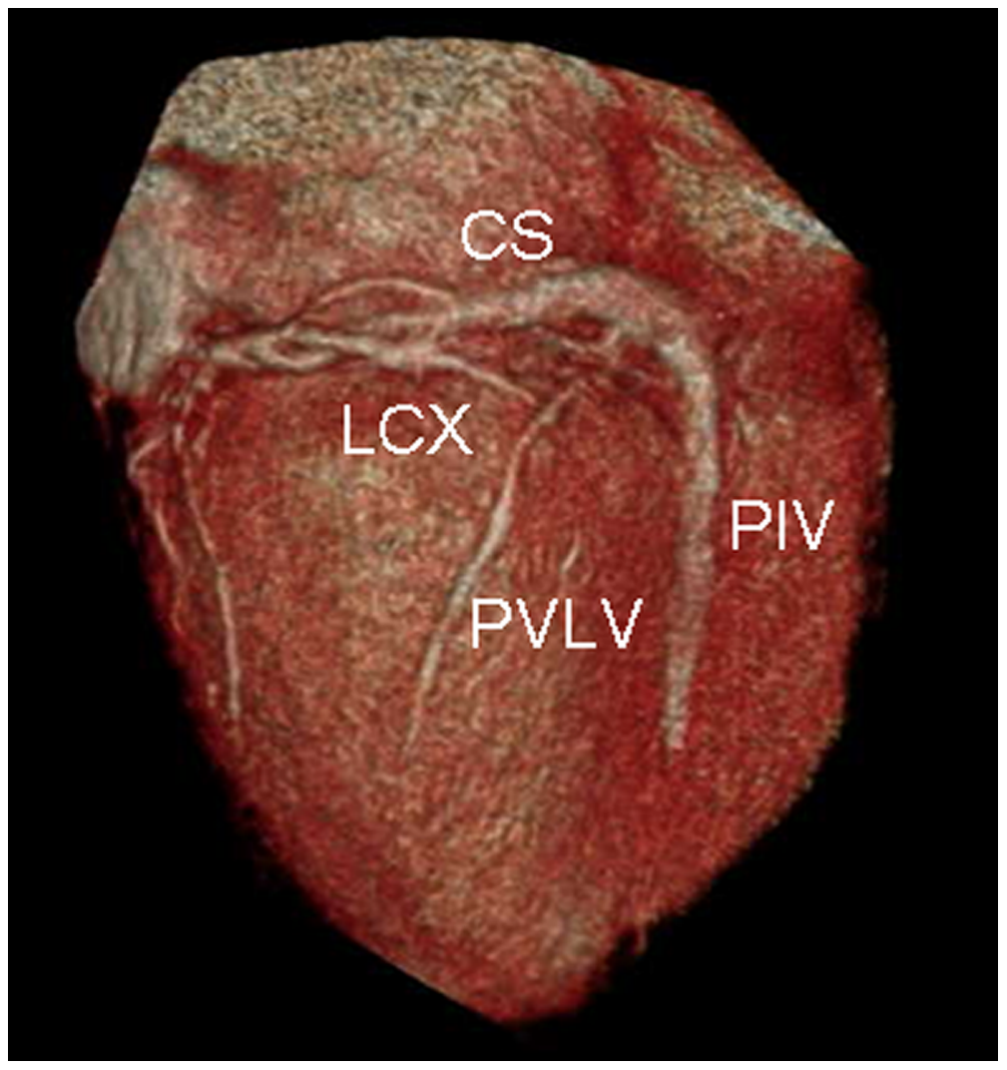
Volume-rendered image shows the spatial relationship of the coronary sinus (CS) and left circumflex artery (LCX). The LCX is interlaced with the CS over a long distance. This close relationship may limit the use of Percutaneous mitral annuloplasty (PMA).

Percutaneous mitral annuloplasty (PMA) has been proposed as an alternative to surgical annuloplasty [21]. With this technique, a double-anchor PMA device is implanted in the CS-GCV at the level of the superior and inferior commissures of the mitral valve. Evaluation of the CS anatomy and its relation to the MVA and the coronary arteries may be of great value in patients who are considered for PMA. In the present study, the CS is located above the level of the MVA in 93.1% of cases. Sometimes, the CS can lie on the left atrial wall for almost its entire course (Figure 10). In 68.6% of the patients, the LCX courses between the CS and the MVA, with a potential risk of compression of the LCX by PMA devices when percutaneous mitral annuloplasty is applied. The close relationship between the CS, coronary arteries, and MVA may result the limits to use PMA, particularly when the artery is interlaced with the CS over a long distance (Figure 11). A surgical approach may be preferred over a percutaneous approach. Knowledge of a detailed anatomic relationship between the CS, coronary arteries, and MVA in humans is essential to define the safety and efficacy of PMA [22].

Radiofrequency catheter ablation (RFA) is a procedure to correct a disturbance in heart rhythm. As a less invasive option to the surgical incisions used in the procedure, such ablation may be performed either by within the chambers of the heart (endocardial ablation) or by outside the heart (epicardial ablation). Epicardial arrhythmias originating from the coronary venous system are usually performed through instrumentation of the CS and its branches. For example, in some idiopathic ventricular tachychardia, coronary veins provide endovascular access to the epicardial regions of the ventricles, especially the crucial area between the junction of the great cardiac vein and the anterior interventricular vein [23]. Ablation can be performed safely from within the coronary venous system. However, coronary artery injury is an ever-present risk, especially near the proximal part of the AIV. A thorough knowledge of the anatomy of veins and arteries prior to the procedure will increase the success rate and will reduce complications.

In addition, the coronary venous system as a route for administering pharmacologic therapy, gene therapy, growth factors, and stem cells to the myocardium has shown higher success rate than intra-arterial or systemic delivery.

### Study limitations

We also acknowledged some limitations in our study. First, the 256-slice CT scans were tailored for optimal visualization of the coronary arteries, which may also be limited in showing the side branches with a small diameter of the coronary veins. Van de Veire et al. [24] reported that an additional 2-second delay was applied after the contrast bolus reached the descending aorta before triggering the scan and the use of saline chasing was omitted for optimal visualization of the coronary venous system. Second, the radiation dose in CT is a limitation that can be modified using dose reduction techniques, for instance, the use of prospective ECG-gating for coronary CTA [25]. Finally, there were no patients with advanced heart failure, atrial fibrillation or mitral valve disease in our study. Most of patients were examined because of a suspicion of CAD, so we did not have the possibility to compare our 256-slice CT technique with the current invasive gold standard, retrograde cardiac venography.

### Conclusion

256-slice CT is a feasible tool for non-invasive evaluation of the coronary venous system and can provide accurate information of the coronary venous system which has important clinical implications.
