# Correction: Assessment of the Coronary Venous System Using 256-Slice Computed Tomography

**DOI:** 10.1371/journal.pone.0113849

**Published:** 2014-11-17

**Authors:** The PLOS ONE Staff

The following information is missing from the Funding section: Funding was received from the National Natural Science Foundation (81401385).
